# The neurotrophic compound J147 reverses cognitive impairment in aged Alzheimer's disease mice

**DOI:** 10.1186/alzrt179

**Published:** 2013-05-14

**Authors:** Marguerite Prior, Richard Dargusch, Jennifer L Ehren, Chandramouli Chiruta, David Schubert

**Affiliations:** 1 The Salk Institute for Biological Studies, Cellular Neurobiology Laboratory, 10010 North Torrey Pines Road, La Jolla, CA 92037, USA

## Abstract

**Introduction:**

Despite years of research, there are no disease-modifying drugs for Alzheimer's disease (AD), a fatal, age-related neurodegenerative disorder. Screening for potential therapeutics in rodent models of AD has generally relied on testing compounds before pathology is present, thereby modeling disease prevention rather than disease modification. Furthermore, this approach to screening does not reflect the clinical presentation of AD patients which could explain the failure to translate compounds identified as beneficial in animal models to disease modifying compounds in clinical trials. Clearly a better approach to pre-clinical drug screening for AD is required.

**Methods:**

To more accurately reflect the clinical setting, we used an alternative screening strategy involving the treatment of AD mice at a stage in the disease when pathology is already advanced. Aged (20-month-old) transgenic AD mice (APP/swePS1ΔE9) were fed an exceptionally potent, orally active, memory enhancing and neurotrophic molecule called J147. Cognitive behavioral assays, histology, ELISA and Western blotting were used to assay the effect of J147 on memory, amyloid metabolism and neuroprotective pathways. J147 was also investigated in a scopolamine-induced model of memory impairment in C57Bl/6J mice and compared to donepezil. Details on the pharmacology and safety of J147 are also included.

**Results:**

Data presented here demonstrate that J147 has the ability to rescue cognitive deficits when administered at a late stage in the disease. The ability of J147 to improve memory in aged AD mice is correlated with its induction of the neurotrophic factors NGF (nerve growth factor) and BDNF (brain derived neurotrophic factor) as well as several BDNF-responsive proteins which are important for learning and memory. The comparison between J147 and donepezil in the scopolamine model showed that while both compounds were comparable at rescuing short term memory, J147 was superior at rescuing spatial memory and a combination of the two worked best for contextual and cued memory.

**Conclusion:**

J147 is an exciting new compound that is extremely potent, safe in animal studies and orally active. J147 is a potential AD therapeutic due to its ability to provide immediate cognition benefits, and it also has the potential to halt and perhaps reverse disease progression in symptomatic animals as demonstrated in these studies.

## Introduction

Alzheimer's disease (AD) is characterized pathologically by the presence of both extracellular neuritic plaques and intracellular neurofibrillary tangles [1]. Clinically, AD results in a progressive loss of cognitive ability as well as daily function activities [2,3]. At the time when most patients are diagnosed with AD, the pathology is usually at an advanced stage. Currently approved therapies are only symptomatic in nature, providing modest improvements in memory without altering the progression of the disease pathology [4,5]. Thus, effective disease modifying treatments that also provide cognition benefits are urgently required.

Age is the greatest risk factor for developing AD, leading us to develop a drug discovery procedure that is based upon old-age-associated pathologies without requiring pre-selected molecular targets [6,7]. A series of six cell culture assays was designed to mimic multiple old-age-associated pathways of central nervous system (CNS) nerve cell damage, and drug candidates were required to show efficacy in all of these assays before being moved forward into animals. As potential lead drug candidates, we generated a large number of derivatives of the curry spice curcumin, which is effective in AD transgenic mice [8,9]. Based upon activity in multiple CNS toxicity assays, we identified an exceptionally potent, orally active, neurotrophic molecule called J147 that facilitates memory in normal rodents, and prevents the loss of synaptic proteins and cognitive decline when administered to three-month-old APP/swePS1ΔE9 mice for seven months [7]. The neurotrophic and memory-enhancing activities of J147 are associated with an increase in the level of brain derived neurotrophic factor (BDNF) along with the expression of BDNF-responsive proteins, the enhancement of long term potentiation (LTP), synaptic protein preservation, the reduction of markers for oxidative stress and inflammation, the reduction of amyloid plaques, and lower levels of soluble Aβ_1-42 _and Aβ_1-40. _These combined neuroprotective and memory enhancing effects of a single molecule suggest that J147 has significant potential for the treatment of AD.

To more closely mimic the clinical setting, we have now examined the effect of J147 in transgenic mice at a stage when pathology is significantly advanced and asked if the drug could rescue some of the symptoms. This study used the well characterized APPswe/PS1ΔE9 mouse model that exhibits a subset of behavioral and pathological features of AD, including age-dependent accumulation of beta-amyloid (Aβ) as well as learning and memory deficits [10]. This model was previously used to demonstrate the neuroprotective and memory enhancing effects of J147 when administered before pathology is present [7]. In comparison, the AD mice in this study were allowed to age to 20 months before being fed J147 for 3 months. We demonstrate that J147 has the ability to rescue the severe cognitive deficits present in aged, transgenic AD mice. In addition, J147 enhances the expression of BDNF and nerve growth factor (NGF) and additional proteins associated with their signaling pathways. Therefore, the reversal of cognitive deficits as well as some other aspects of AD pathology by J147 may result from an up-regulation of BDNF and NGF pathways.

## Materials and methods

### Materials

High glucose Dulbecco's modified Eagle's medium (DMEM) and fetal calf serum (FCS) were obtained from Invitrogen (Carlsbad, CA, USA). C57BL/6J mice were ordered from Jackson Labs (Sacramento, CA, USA) Stock 000664. The transgenic mouse line APPswe/PS1ΔE9 85 was a generous gift of Dr. J.L. Jankowsky.

The primary antibodies were used at a dilution of 1:1,000 unless otherwise stated and their sources and molecular weights were as follows: Cell Signaling Technology (Danvers, MA, USA): β-actin, monoclonal HRP conjugate, 45 kDa; CREB, monoclonal, 43 kDa. Santa Cruz Biotechnology (Santa Cruz, CA, USA): Egr-3, C-24 polyclonal, 42 kDa; BDNF, polyclonal, 16 kDa. Millipore (Temecula, CA, USA): Anti-BACE C-terminus, clone 61-3E7, 60 to 75 kDa. Novus Biologicals (Littleton, CO, USA): Homer-1, polyclonal, 47 kDa. Sigma (St Louis, MO, USA): Anti-Amyloid Precursor Protein, C-terminal, polyclonal, 95 to 100 kDa; Anti-Nerve Growth Factor 2.5S, polyclonal homodimer, 26 kDa. Covance (Princeton, NJ, USA): 6E10 monoclonal antibody.

All other materials were from Sigma (St Louis, MO, USA) unless otherwise stated.

### Methods

#### Animal studies

All animal studies were carried out in strict accordance with the recommendations in the Guide for the Care and Use of Laboratory Animals of the National Institutes of Health. The protocol was approved by the Committee on the Ethics of Animal Experiments of the Salk Institute for Biological Studies.

### Old huAPPswe/PS1ΔE9 transgenic mice

#### Animals

The APPswe/PS1ΔE9 transgenic mice (line 85) have been previously characterized [10,11]. The line 85 mice carry two transgenes, the mouse/human chimeric APP/Swe, linked to Swedish FAD and human PS1ΔE9. At 20 months of age both male and female transgenic mice were fed a high fat diet (Harlan Tekland, Madison, WI, USA) with and without J147 (200 ppm, 10 mg/kg/day). Treatment continued for three months and was followed by behavior testing and sacrifice of mice for tissue harvesting. Mouse body weights and food consumption were measured weekly, and there were no significant differences between the groups. (Data not shown).

### C57BL/6J mice-scopolamine study

#### Animals

A total of 60 male mice aged eight weeks were housed 4 per cage and were divided into five groups with 12 mice per group. Treatments were administered in the food (TestDiet^® ^5015, Richmond, IN, USA) for a period of two weeks before commencement of behavioral testing. Groups included J147 at 200 ppm (10 mg/kg/day), donepezil at 20 ppm (1 mg/kg/day), a combination of J147 at 200 ppm and donepezil at 20 ppm, and two groups on the control food without any treatments. Following two weeks of treatment, memory impairment was induced by intraperitoneal (i.p.) injection of scopolamine (1 mg/kg) 30 minutes prior to each of the following behavioral tests: Y-maze, probe trial of the water maze and contextual and cued fear conditioning. Mice were allowed to rest for two days between each behavior test. All mice received scopolamine except for one of the control groups, which received saline as a control. Mice were sacrificed 24 hours after the last behavioral test for tissue harvesting.

### Behavior assays

#### Two-day water maze

Spatial memory was determined using the two-day water maze in 23-month-old huAPPswe/PS1 transgenic mice fed J147 at 200 ppm in food for the previous three months. The protocol was adapted from a publication by Gulinello and colleagues [12]. Water temperature remained at 27°C throughout the experiment. The goal platform was positioned 45 cm from the outside wall in the north-west quadrant of the maze for all groups and all trials. Day 1 of the two-day water maze procedure involved training the mice to find the platform using cues located around the pool within a 180 s time frame. This training involved a series of visible platform trials where mice were tracked using the Noldus EthoVision software (Noldus Information Technology, Inc., Leesburg, VA, USA). There were four visible platform trials (V1 to V4) where the last visible platform trial of a mouse was considered its post-habituation baseline. If the mice failed to find the platform after 180 s, they were placed on the platform by the experimenter. All mice remained on the platform for 15 s before being placed in a heated incubator (30°C) between trials. On Day 2, 24 hours following the last visible platform trial, mice were tested in a series of three hidden platform trials (T1 to T3). Again each trial lasted for 180 s. The time it took each mouse to find the hidden platform was measured as escape latency. For the scopolamine experiment, normal mice were given an i.p. injection of saline or 1 mg/kg scopolamine 30 minutes before the first hidden platform trial on Day 2. All trials were recorded using the EthoVision Software and statistics were computed using GraphPad Instat software (GraphPad Software, San Diego, CA, USA).

#### Elevated plus maze

The elevated plus maze analyzes the anxiety response of mice [13]. This test relies upon the tendency of mice to have a fear of heights and to navigate towards dark enclosed spaces and remain there [14]. Our maze is made of gray plastic and consists of four arms (two open without walls and two enclosed by 15.25 cm high walls) 30 cm long and 5 cm wide in the shape of a plus sign. The elevated plus maze is placed close to the center of the room, and has similar levels of illumination on both open and closed arms. A video-tracking system (Noldus EthoVision) is used to automatically collect behavioral data. The software is installed on a PC computer with a digital video camera mounted overhead on the ceiling, which automatically detects and records when mice enter the open or closed arms of the maze and the time spent in each. Mice are habituated to the room 24 hours before testing. Mice are also habituated to the maze for two minutes before testing by placing them in the center of the maze and blocking entry to the arms. Mice were then tested in the maze for a five-minute period while the software tracked and recorded the behavior of the mice. The anxiety of mice was measured by comparing the time spent in the open arms to time spent in the closed arms. Statistics were computed using GraphPad Instat software.

#### Fear conditioning assay

Fear conditioning to either a cue or a context represents a form of associative learning. The read-out that is measured in contextual and cued fear conditioning is a freezing response that occurs following the pairing of an unconditioned stimulus (US), such as a foot shock, with a conditioned stimulus (CS), such as a particular context or cue (tone) [15-17]. The mouse will freeze if it remembers and associates that environment with the aversive stimulus. The hippocampus and the amygdala are required for fear memory where the hippocampus is involved in the formation and retrieval of context fear associations and the amygdala is involved in conditioning and recall of associations to contextual and discrete cues [18,19]. This assay used fear conditioning chambers from Med Associates Inc. with Video Freeze Software (Med Associates Inc, St. Albans, VT, USA). On Day 1, mice were trained by allowing them to explore the chamber for 120 sec, mice were then presented with a 30-sec tone (2 kHz with 85 dB intensity) immediately followed by a 2-sec foot shock (0.7 mA). The tone-shock pairing was repeated following a 30-sec interval and the mice were again allowed to explore for 120 sec before removing them from the chamber. On Day 2, contextual memory, which requires a functioning hippocampus, was tested by placing the mice in the chambers and allowing them to explore for the same length of time as the previous day but without the tone and the shock. On Day 3, cued or emotional memory was tested, which relies on both hippocampus and amygdala. For this, the chamber environment was altered by using plastic boards to alter the shape of the chamber and using similar plastic boards over the grid floor to alter the environment further. Vanilla essence was used to alter the smell of the environment. Testing involved placing the mice in the chambers and carrying out the same paradigm as Day 1 without the foot shock. The camera measures the amount of time the mice freeze and the software allows analysis of this freezing at any time-point of interest. On Day 2, the time spent freezing is measured over the entire time in the chamber. A mouse that remembers the chamber context and associates it with the foot shock will spend more time freezing and this response is hippocampal dependent. The percentage of time spent freezing by each mouse is averaged per group and then groups can be compared and *P*-values calculated to determine statistical significance. On Day 3, the percentage of time spent freezing during the two tones is averaged per group, and then groups can be compared and *P*-values calculated to determine statistical significance. This result relates to the recall of associations to the tone and is dependent on the amygdala and the hippocampus. For the scopolamine experiment, normal mice were administered an i.p. injection of saline or 1 mg/kg scopolamine 30 minutes before testing on Day 2 and Day 3.

#### Y-Maze

Spontaneous alternation, the tendency to alternate free choices in a Y-maze (three arms), is a model for studying short term working memory in mice [20,21]. Mice were injected with 1 mg/kg of scopolamine or saline 30 minutes prior to testing. Then each mouse was placed in the center of the Y and arm entries were recorded by video camera and the order of entries were recorded for the first 15 entries. Spontaneous alternations are defined as consecutive triplets of different arm choices.

### Tissue preparation and immunoblotting

Hippocampal and entorhinal cortex tissue samples were homogenized in 10 volumes of RIPA lysis buffer (50 mM Tris, pH 7.5, 150 mM NaCl, 0.1% sodium dodecyl sulfate and 0.5% deoxycholate, and 1% NP40) containing a cocktail of protease and phosphatase inhibitors (20 mg/ml each of pepstatin A, aprotinin, phosphoramidon, and leupeptin; 0.5 mM 4-(2-aminoethyl) benzenesulfonyl fluoride hydrochloride; 1 mM EGTA; 5 mM fenvalerate; and 5 mM cantharidin). Samples were sonicated (2 × 10 s) and centrifuged at 100,000 × g for 60 minutes at 4°C. Protein concentrations in the cell extracts were determined using the BCA protein assay (Pierce supplied by Thermo Fisher Scientific, Rockford, IL, USA). Equal amounts of protein were solubilized in 2.5x SDS-sample buffer, separated on 12% SDS-polyacrylamide gels, transferred to Immobulin P and immunoblotted with the antibodies indicated in the Materials section. For Western blots, protein levels were normalized to actin levels. An unpaired *t *test was performed to compare between two groups at a single time point. When comparing multiple groups, one-way ANOVA followed by a Tukey's *post hoc *test was used. All statistical analysis was conducted using GraphPad Instat software.

### Immunohistochemistry

Brains were fixed with 4% paraformaldehyde in 100 mM sodium tetraborate, pH 9.5, for 3 h, cryoprotected with 20% sucrose-potassium-PBS (KPBS), and sectioned into coronal (30 μm) sections using a sliding microtome (Leica Microsystems Inc, Buffalo Grove, IL, USA). Sections were submerged in 0.3% H_2_O_2 _for 10 minutes to eliminate endogenous peroxidase activity and treated with 1% borate to eliminate free paraformaldehyde. Sections were incubated with primary antibody in 0.3% Triton X-100 in KPBS plus 2% filtered serum or BSA overnight at 4°C, and with primary antibodies (1:1,000) in 0.3% Triton X-100 for 1 hr at room temperature. After incubation with secondary antibody and ABC reagent (Vector Laboratories Inc, Burlingame, CA, USA), sections were developed using metal-enhanced DAB solution. Sections were mounted to slides, dried, dehydrolyzed, treated with xylene, and covered using permount (Fisher Scientific, Pittsburgh, Pennsylvania, USA). Images were captured by a Zeiss digital camera connected to a Zeiss VivaTome microscope (Carl Zeiss Microscopy, LLC, Thornwood, NY, USA), and image analysis on sections was performed using Axiovision software (Carl Zeiss Microscopy, LLC, Thornwood, NY, USA).

Quantification of amyloid plaque burden was based on the image captured by immunohistochemical staining with antibody 6E10. Sections of each mouse cortex and hippocampus were imaged together and the areas and densities of the plaques in the hippocampus only were measured by the Image J software (NIH). The total counts of Aβ plaques in sections per six mouse brains of each group were determined in an unbiased fashion.

### Aβ ELISA

Aβ 1 to 40 and 1 to 42 levels in hippocampal lysate were analyzed using the Aβ_1-40 _and Aβ_1-42 _ELISA kits from Invitrogen (# KHB3481 and # KHB3442, respectively). All kit reagents were brought to room temperature before use. Standards were prepared according to the manufacturer's guidelines and samples were diluted as follows; RIPA fractions were diluted 1:10 for both Aβ_1-40 _and Aβ_1-42_; and RIPA insoluble fractions were diluted 1:2,000 for Aβ_1-40 _and 1:5,000 for Aβ_1-42_. A total of 50 μl of Aβ peptide standards and samples were added in duplicate to 96-well plates pre-coated with mAb to the NH_2 _terminus region of Aβ. Plates were incubated at 4°C overnight and then 50 μl of Hu Aβ40 or Aβ42 detection antibody was added to each well except the chromogen blanks. Plates were incubated at room temperature with gentle shaking for three hours and then washed four times with the provided wash buffer. At this time, 100 μl of anti rabbit IgG HRP working solution was added to each well except the chromogen blanks for 30 minutes at room temperature. Wells were then washed as before four times and incubated with 100 μl of stabilized chromogen for 25 minutes at room temperature in the dark. Stop solution was then added at 100 μl to each well followed by reading the absorbance of each well at 450 nm. Curve fitting software was used to generate the standard curve where a four-parameter algorithm provided the best standard curve fit. The concentrations of the samples were calculated from the standard curve and multiplied by the dilution factor.

### Cell culture with growth conditioned medium

The HT22 cell line was used to make growth conditioned medium. HT22 is a nerve cell line derived from mouse brain and is widely used to study nerve cell physiology [22,23]. To make HT22 growth conditioned medium, cells were grown in DMEM with 10% fetal calf serum. Then, semiconfluent cultures were washed three times with serum-free DMEM and cultured overnight in a reduced volume of DMEM in the presence or absence of 100 nM J147. The following day, the growth conditioned medium was collected and centrifuged at 10,000 × g to remove detached cells. To determine the effect of the conditioned medium on NGF-induced neurite outgrowth, PC12 cells were dissociated and plated on polyornithine-coated tissue culture dishes in the following conditions: 1) HT22 conditioned medium, 2) J147 treated HT22 conditioned medium, 3) DMEM alone plus J147, 4) DMEM plus NGF at 50 nanograms per ml, 5) J147 treated HT22 conditioned medium pre-incubated for one hour with 10 μg/ml anti-NGF and N2 supplement (Invitrogen). N2 supplement, which contains transferrin, was used in the presence of antibody to minimize the possibility that antibody protein non-specifically modified cell-substrate from adhesion and therefore neurite outgrowth. Phase contrast photographs were taken 24 hrs later.

### GeneChip

#### HT22 cells

HT22 cells were plated in DMEM plus 10% FCS. The next day, the cells were treated with 10 μM J147 for 1 hr before RNA isolation.

#### RNA isolation

RNA was isolated with the use of RNeasy Mini kit (Qiagen, #74104; Valencia, CA, USA) according to the manufacturer's instructions. Total RNA was quantified using the ND-1000 Nanodrop and assessed for quality using the ratios: A260/280 (range: 1.9 to 2.1) and A260/230 (range: 2.0 to 2.2, if <2.0, contamination), in addition to the Bioanalyzer (Agilent Technologies, Cedar Creek, TX, USA) if further quality assessment was required.

#### RNA isolation and microarray hybridization experiments

After RNA isolation for each sample, double stranded cDNA was synthesized from 500 ng total RNA and biotin-labeled using the GeneChip 3' IVT Express Kit (Affymetrix, Santa Clara, CA, USA, #901228-A) per the manufacturer's instructions and protocol. RNA was purified, quantified, fragmented randomly to an average size of 50 to 200 bases and hybridized to GeneChip^® ^Mouse Genome 430 2.0 Arrays (Affymetrix,) consisting of over 45,000 probe sets representing more than 34,000 named mouse genes. The hybridization and processing of the GeneChips were conducted by Salk Institute's Functional Genomics Core Facility using the following systems from Affymetrix (Santa Clara, CA, USA): GeneChip^® ^Hybridization Oven 640, GeneChip^® ^Fluidics Station 450 to the wash and stain operation of Affymetrix GeneChip^® ^arrays, and the GeneChip^® ^Scanner 3000 7G.

#### GeneChip quantification and normalization

Affymetrix Expression Console Software (version 1.0) was used to perform quality assessment of the microarray scan/experiments. Array data were normalized via scaling to adjust the average intensity of each array to be similar. GeneChips were analyzed by the GeneChip Operating Software (Affymetrix) with the default settings except that the target signal was set to 200 for GeneChip quality control. Raw data were analyzed through the gcRMA-algorithm using the Affymetrix package in R software for statistical computing and graphics [24]. The median microarray intensity for all microarrays was normalized to 100, and probe sets with median intensities >100 were scored. Fold changes were calculated in Microsoft Excel Microsoft, Redmond, Washington, USA). Genes of interest and genes with the highest fold-changes were validated using Real-Time Quantitative PCR. The data discussed in this publication have been deposited in NCBI's Gene Expression Omnibus [25] and are accessible through GEO Series accession number GSE45534 [26].

### Commercial screening for molecular targets and "off target" effects

All screening was done at 10 μM J147 by various contract research organizations (CROs), including MDS Pharma Services (King of Prussia, PA, USA), Ricerca, now Panlabs (Concord, OH, USA), Ambit (La Jolla, CA, USA), Caliper (Hopkinton, MA, USA) and NovaScreen Biosciences (Hanover, MD, USA) by standard protocols described in their catalogs. The only two assays that yielded greater than 60% inhibition at 10 μM J147 were then re-assayed to determine EC_50 _values: the dopamine transporter (EC_50 _= 0.649 μM) and monoamine oxidase B (EC_50 _= 1.88 μM) assays both carried out by MDS Pharma Services.

### Synthesis of J147 and donepezil

#### Materials

Compounds J147 and donepezil were synthesized in our laboratory at the Salk Institute. All starting materials, chemicals and reagents were obtained from Sigma Aldrich, (Milwaukee, WI, USA), and were used as received. Solvents used for synthesis and chromatographic analysis were HPLC or ACS reagent grade and were purchased from Fisher Scientific Co (Pittsburg, PA, USA). Thin layer chromatography (TLC) used EMD silica gel F-254 plates (thickness of 0.25 mm). Flash chromatography used EMD silica gel 60, 230 to 400 mesh and were purchased from EMD Chemicals (San Diego, CA, USA).

#### Analytical methods

^1^H NMR recorded at 500, on a Varian VNMRS-500 spectrometer at the Salk Institute (La Jolla, CA, USA) using the indicated solvents. Chemical shift (*δ*) is given in parts per million (ppm) relative to tetramethylsilane (TMS) as an internal standard. Coupling constants (*J*) are expressed in hertz (Hz), and conventional abbreviations used for signal shape are as follows: s = singlet; d = doublet; t = triplet; m = multiplet; dd = doublet of doublets; brs = broad singlet. Liquid chromatography mass spectrometry (LCMS) was carried out using a Shimadzu LC-20AD spectrometer at The Scripps Research Institute (La Jolla, CA, USA), and electrospray ionization (ESI) mass analysis with a Thermo Scientific LTQ Orbitrap-XL spectrometer at the Salk Institute. Melting points were determined with a Thomas-Hoover capillary melting point apparatus at the Salk Institute, and are uncorrected. All final compounds were characterized by LCMS and ^1^H NMR and gave satisfactory results in agreement with the proposed structure. J147 and donepezil have purity of 98% and 99%, respectively, which was determined by analysis on a C18 reverse phase HPLC column (Phenomenex Luna (50 mm × 4.60 mm, 3 μm)) at The Scripps Research Institute, using 10 to 90% CH_3_CN/H_2_O containing a 0.02% AcOH with a flow rate of 1 mL/min (5-minute gradient) and monitoring by a UV detector operating at 254 nm.

### Chemical synthesis of compounds

The synthesis of J147 has been carried out using simple chemistry as described in our previous paper by condensation of 3-methoxybenzaldehyde and (2, 4-dimethylphenyl) hydrazine hydrochloride in EtOH at room temperature, followed by acetylation using trifluoroacetic anhydride and triethylamine in CH_2_Cl_2 _gave J147 (Scheme 1). Donepezil has been synthesized with 99% purity according to the literature procedure published in *Organic Process Research & Development *2008, **12**:731-735 (Scheme 2).

### Synthesis of (E)-N-(2,4-dimethylphenyl)-2,2,2-trifluoro-N'-(3methoxybenzyli-dene) acetohydrazide (J147)

A mixture of 3-methoxybenzaldehyde (50 g, 367.64 mmol) and (2, 4-dimethylphenyl) hydrazine hydrochloride (63.23 g, 367.64 mmol) in EtOH (50 mL) was stirred at room temperature for 1 h, the obtained solid was filtered off, washed with ethanol and dried under vacuum to afford hydrazone hydrochloride 1 (95.94 g) in 90% yield as a light brown solid. This unstable hydrazone (50 g, 172.41 mmol) was dissolved in CH2Cl2 (50 ml), Et3N (57.56 mL, 413.79 mmol) followed by (CF3CO)2O (28.77 mL, 206.89 mmol), was added at 0°C and the mixture was stirred at room temperature for 1 h. Reaction mixture was diluted with aq. sat. NaHCO3 solution (500 mL), extracted with CH2Cl2 (2 × 500 mL), dried (Na2SO4) and evaporated, resulting solid was recrystalized from ethanol to give J147 (49.11 g, 81%) as a white solid: mp 70 to 72°C; LCMS purity 98%; ^1^H NMR (CDCl3, 500 MHz) *δ *ppm 2.10 (s, 3H), 2.42 (s, 3H), 3.82 (s, 3H), 6.98 (dd, *J *= 8.5, 2.0 Hz, 1H), 7.07 (d, *J *= 7.5 Hz, 1H), 7.14 (d, *J *= 8.0 Hz, 1H), 7.28 (m, 3H). MS (ESI): *m/z *calcd for C18H17F3N2O2 ((M + H) ^+^) 351.1314; found 351.1366 ((M + H) ^+^).

## Results

The early intervention strategy is most frequently used for drug testing in AD because drug candidates assayed in AD transgenic mice at the stage when pathology is already present are generally less effective [27-30]. However, treating aged, transgenic AD mice in an AD reversal type investigation is a valuable experiment that should provide important pre-clinical information about the ability of compounds to rescue cognitive deficits in AD patients. We used 20-month-old line 85 APPswe/PS1ΔE9 transgenic AD mice (huAPP/PS1) to examine the effect of J147 on the physiological, behavioral and pathological aspects of the disease when administered late in the course of the disease. Pathological hallmarks of AD, including plaques and loss of synaptic markers, are readily apparent at nine months of age in huAPP/PS1 mice along with clear deficits in spatial memory when compared to wild type controls [7,10,11,31]. Wild type controls were not included in this study because deficits in huAPP/PS1 mice compared to wild type controls have already been extensively characterized and documented in the literature [7,10,11,31].

### J147 rescues cognitive deficits in aged Alzheimer's disease mice

**Figure 1:**
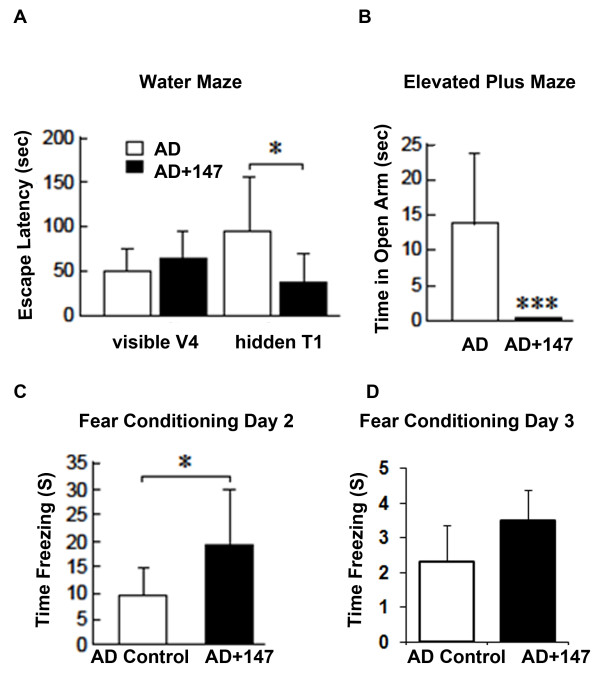
**J147 rescues cognitive deficits in aged Alzheimer's disease mice**. huAPP/PS1 mice were aged to 20 months old. The mice were then put on the control food diet or 200 ppm J147 food diet (10 mg/kg/day). Following three months of treatment, all mice were analyzed for spatial memory by the two-day water maze, disinhibition phenotype by the elevated plus maze and hippocampal dependent memory by fear conditioning. **(A) **The two-day water maze analyzes spatial navigational memory. Visible V4 refers to visible platform trial 4 (Day 1), which is the last visible platform trial before testing and, therefore, represents the baseline. During testing on Day 2, the time it takes each mouse to find the hidden platform during trial 1 (hidden T1) is measured as escape latency. AD mice control (white boxes), took considerably longer to find the hidden platform than AD mice fed J147 (black boxes), suggesting that J147 improved the navigational memory of mice. **(B) **The elevated plus maze analyzes the anxiety response of mice by comparing the time spent on the open arms to time spent on the closed arms. AD alone mice (white box) spent more time in the open arms than AD mice fed J147 (black box) suggesting that J147 treatment reduced the disinhibition phenotype. **(C **and **D) **Fear conditioning to either a cue or a context represents a form of associative learning. The readout that is measured in contextual and cued fear conditioning is a freezing response that occurs following the pairing of an unconditioned stimulus such as a foot shock, with a conditioned stimulus such as a particular context or cue (tone). (C) AD mice on the J147 diet (black box) spent much more time freezing on Day 2, demonstrating a significant improvement in hippocampal related memory compared to AD mice on the control diet (white box). (D) There was no significant difference in freezing response on day three (cued memory) between AD control and AD mice on J147 diet. These results suggest that J147 improved the cognitive performance in AD mice. One-way ANOVA and Tukey *post hoc *test were used to determine the statistical significance of the behavioral responses, N = 11 AD Control, N = 12 AD + J147. *P <0.05, *** *P *<0.001.

Both male and female huAPP/PS1 mice were aged to 20 months. At this time, the mice were randomly assigned to one of two groups: 11 mice were fed normal food and 13 mice were fed the same diet but also containing 200 ppm J147. This concentration of J147 converts to approximately 10 mg/kg/day per mouse and was chosen based upon the potency of J147 in our recently published study [7]. Following three months of treatment, all mice were analyzed for spatial memory performance by the two-day water maze (Figure 1A), disinhibition phenotype by the elevated plus maze (Figure 1B), and contextual and cued memory by a fear conditioning assay (Figure 1C and 1D, respectively). There were no significant differences between the sexes within each group.

The two-day water maze analyzes spatial navigational memory [12], which is impaired in huAPP/PS1 mice when compared to wild type littermates [10,32]. This water maze differs slightly from the traditional Morris Water Maze which involves a five- to seven-day training period to analyze learning and a final probe day to analyze memory [33]. Briefly, a platform which is visible during training on Day 1 is then submerged just under the water level during testing on Day 2 and mice use spatial cues on the wall around the pool to navigate to the platform during testing. In Figure 1, visible V4 refers to visible platform trial 4 (Day 1), which is the last visible platform trial before testing and, therefore, represents the baseline. Results from Day 1 indicate no defects in AD or AD + J147 in the ability to swim or see as both have similar escape latency. During testing on Day 2, the time it takes each mouse to find the hidden platform during trial 1 (hidden T1) is measured as escape latency. Results from this two-day water maze show that AD mice take considerably longer to find the hidden platform on Day 2 than AD mice treated with J147 for three months (Figure 1A), demonstrating that J147 significantly improved the spatial navigational memory in aged, transgenic AD mice.

There is a growing body of evidence that dementia is clinically associated with anxiety [34]. The elevated plus maze measures the anxiety response of mice [13] by comparing the time spent in the open arm to the time spent in the closed arm. Anxiety behavior is affected in transgenic AD mouse models but the results vary considerably according to strain, age and the model used for the study. AD mice tend to exhibit a disinhibition phenotype and will spend more time in the open arm than in the closed arm [35-38]. The level of anxiety in APPswePS1ΔE9 mice was increased according to one report [39] whereas in 7-month- and 12-month-old mice of the same strain, there was a reduction in anxiety compared to wild type controls [40,41]). A reduction in anxiety represents a disinhibition phenotype that may be viewed as akin to that reported in some patients with Alzheimer's disease, exemplified by socially unacceptable behaviors [42]. In addition to the strain and age differences, variation in the methodology and the laboratory conditions could interfere with the anxiety and exploration behavior in rodents [43]. These variables could explain observed differences in results. Our data demonstrate that aged transgenic AD mice do indeed spend more time in the open arm, a phenotype that was completely rescued by treatment with J147 for three months (Figure 1B).

Fear conditioning measures hippocampal-dependent associative learning. The read-out measured is a freezing response where the mouse will freeze if it remembers and associates that environment with the aversive stimulus. The hippocampus and the amygdala are required for fear memory [18,19]. Contextual fear conditioning has previously been carried out with the huAPP/PS1 mice where 11-month-old AD mice spent significantly less time freezing in response to the context than wild type controls [44]. AD mice alone spent significantly less time freezing in response to the context associated with the aversive stimulus in our experiment, indicating that they did not remember the context, a phenotype that was rescued by treatment with J147 (Figure 1C). There was no significant difference between the groups on Day 3 of the assay suggesting that the amygdala was not affected by J147 treatment (Figure 1D).

Results from these behavioral assays show that J147 has the ability to rescue the cognitive decline and disinhibition phenotype associated with AD when administered at an extremely late stage in the disease progression when pathology is already far advanced.

### J147 reduces soluble levels of Aβ

**Figure 2:**
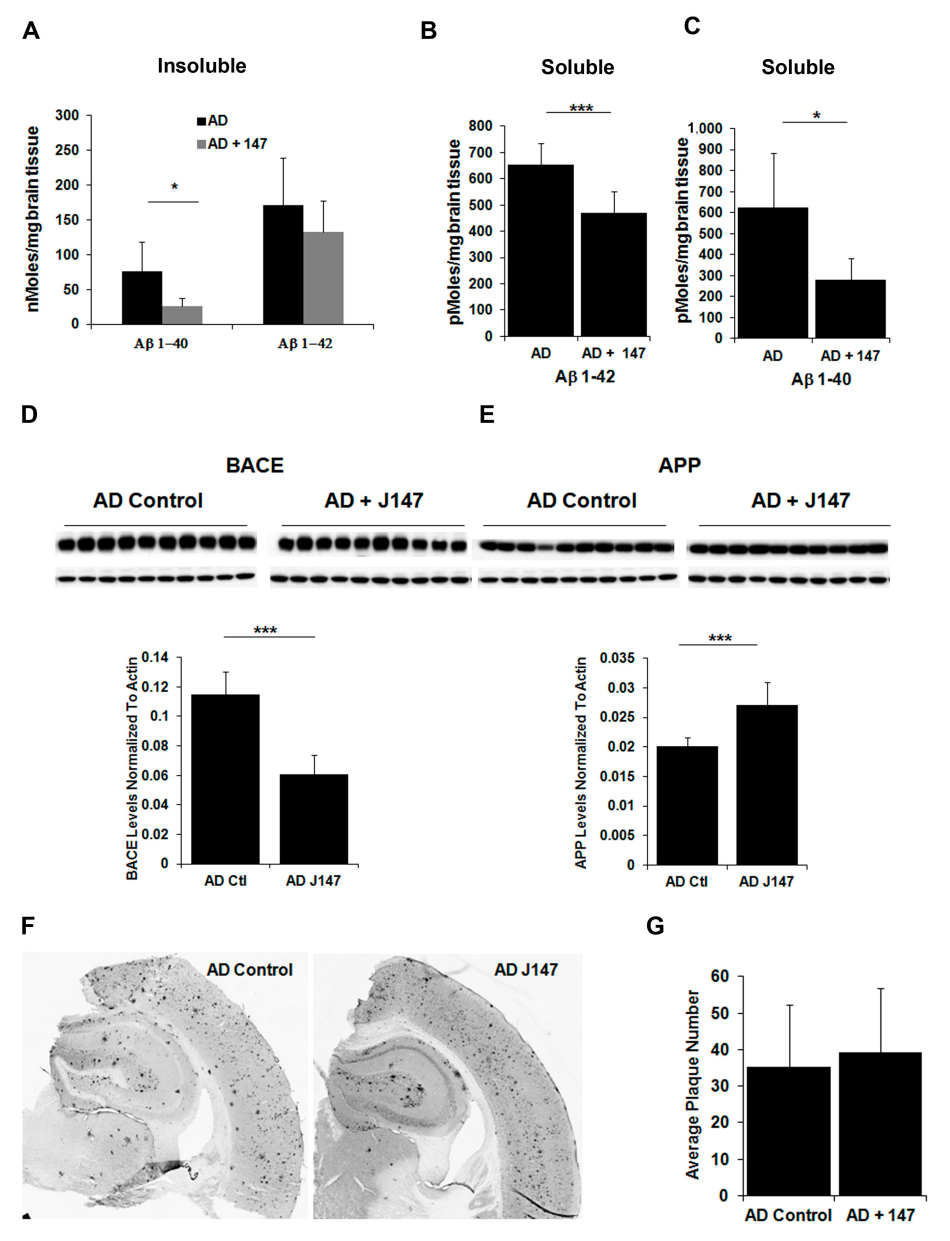
**J147 reduces soluble levels of Aβ**. Hippocampal tissue from the aged huAPP/PS1 mice was analyzed for the effect of J147 treatment on Aβ levels. Aβ_1-40 _and Aβ_1-42 _levels were measured by ELISA in control AD animals (black bars) and AD animals fed J147 (grey bars) in the insoluble (100,000 xg pellet) **(A) **and RIPA soluble fractions **(B **and **C)**. J147 treatment decreased insoluble Aβ_1-40 _and both soluble Aβ_1-40 _and Aβ_1-42 _levels. Cell lysates of hippocampal tissue from the aged huAPP/PS1 and control mice were analyzed for an effect of J147 on the APP processing pathway by immunoblotting with antibodies against BACE **(D) **and APP **(E)**. The images were quantified and are represented in the bar graphs accompanying the Western blot images (D and E). BACE levels were significantly reduced upon treatment with J147 with a corresponding significant increase in APP levels. Two-tailed *P-*values ***P <0.001. All data shown are means ± SD, *n *= 10 to 11 per group. **(F) **Immunohistochemical analysis was done using brain coronal sections from these same mice with the antibody 6E10. Sections (30 μ thick) of similar regions from each mouse, (N = 6) were examined and plaque counts in the hippocampus were quantified. All immunohistochemical images were quantified using Image J Software. **(G) **The average of plaque counts for each mouse group is expressed as a number of plaques ± the SD.

Since it is now believed that soluble Aβ polymers are major contributors to the toxicity associated with the peptide [45,46] and soluble Aβ is the primary contributor to cognitive dysfunction in line 85 huAPP/PS1 mice [46], we examined Aβ levels in the RIPA insoluble (100,000 × g pellet) and soluble (RIPA supernatant) fractions of the hippocampus of J147-treated and control huAPP/PS1 mice. While Aβ_1-42 _levels as measured by ELISA were not altered in the RIPA insoluble fraction in animals fed J147 relative to untreated AD transgenic animals, Aβ_1-40 _levels were reduced in this fraction (Figure 2A). Figure 2B, C shows that treatment with J147 decreased the amount of RIPA soluble Aβ_1-42 _and Aβ_1-40_, respectively, in the hippocampus of aged huAPP/PS1 mice treated with J147 for three months. Thus, J147 has a small but significant effect on Aβ metabolism by reducing both Aβ_1-40 _and Aβ_1-42 _in the soluble fraction of the hippocampus. Given this effect of J147 on Aβ metabolism, we investigated the effect of J147 on the amyloid precursor protein (APP) processing pathway that leads to the production of Aβ (Figure 2D, E).

The protein level of β-secretase (BACE) in the RIPA soluble fraction from the hippocampus of J147-treated mice is significantly reduced compared to the untreated AD mice (Figure 2D) suggesting that J147 treatment down-regulated BACE, which is critical for APP cleavage that eventually gives rise to Aβ [47-51]. This result is supported by the finding that at the same time that BACE levels are decreased in the hippocampus, APP protein levels are significantly increased in the soluble fractions of hippocampus from the J147-treated AD mice compared to control AD mice (Figure 2E). Lower levels of BACE are consistent with a reduction in cleavage of the substrate, APP, thereby explaining the increased levels of APP. Studies indicate that APP is likely to play a direct role in synaptic structure and function [52,53].

Since some compounds that reduce memory loss in AD mice reduce the Aβ plaque burden, we next examined plaque size and density in the hippocampus of J147-treated and control aged huAPP/PS1 mice. There was no difference in either plaque number or size between control and J147-treated animals (Figure 2F, G). Therefore, while treatment with J147 enhanced the cognitive ability of the huAPP/PS1 mice, it had no significant effect on plaque load but produced a significant reduction of soluble Aβ levels. This reduction of soluble Aβ levels may be due to the effect of J147 on the APP processing pathway by down-regulation of BACE.

### Neuroprotective pathways targeted by J147

J147 was synthesized and selected for its neurotrophic ability, in part using assays where it replaced BDNF function [7]. J147 increases the levels of BDNF in the hippocampus of normal rats, as well as in huAPP/PS1 transgenic mice [7], and its synthetic precursor, CNB-001, increases BDNF levels in rat traumatic brain injury models [54]. J147 also modulates the expression and/or phosphorylation of downstream targets of BDNF [7]. Given these previous findings in relation to the BDNF pathway following J147 treatment, the effect of J147 on neurotrophic factors was investigated in this AD reversal study.

Two of the most prominent members of the mammalian neurotrophin family are BDNF and NGF. These neurotrophic factors stimulate the differentiation, growth and maintenance of developing neurons in addition to the survival of mature neurons, and are key players in synaptic plasticity [55,56], cognition and memory formation [57-62]. These versatile proteins are up-regulated in response to neuronal injury and play a role in the healing process as well as neurogenesis [63,64]. Neurotrophins are synthesized as proneurotrophins, which may have either neurotrophic or pro-apoptotic activity [65].

**Figure 3:**
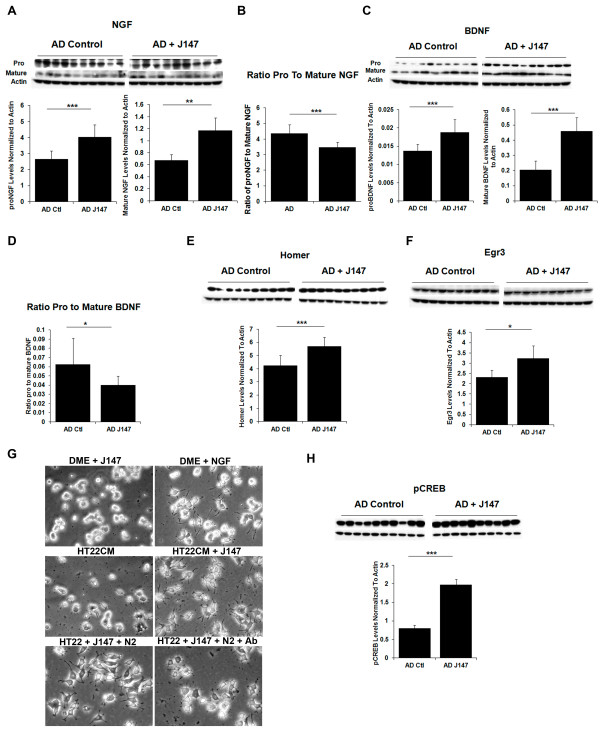
**Neuroprotective pathways targeted by J147**. Cell lysates of either hippocampal tissue or entorhinal cortex tissue from aged AD mice on control diet (AD Ctl) or J147 diet (AD J147) were analyzed by Western blotting and the images quantified in bar graphs accompanying the images. Actin was used as a loading control and all proteins were normalized to actin for quantification purposes. Protein expression levels of both pro- and mature NGF **(A) **are up-regulated in the hippocampus of aged huAPP/PS1 mice fed J147 in their diet for three months compared to control treated mice. **(B) **The ratio of pro- to mature NGF is decreased in aged huAPP/PS1 mice treated with J147. **(C) **Levels of another neurotrophic factor, BDNF, both pro and mature, are also increased in the hippocampus of J147 treated aged huAPP/PS1 mice. **(D) **The ratio of pro- to mature BDNF is decreased in aged huAPP/PS1 mice treated with J147. **(E) **The BDNF responsive protein Homer-1 is also increased in the hippocampus upon treatment with J147 as did Egr3 **(F) **another target gene of BDNF. **(G) **J147 stimulates neurite outgrowth promoting factor. PC12 cells were plated in growth conditioned medium (CM) prepared from HT22 cells incubated overnight plus or minus 100 nM J147, and as controls fresh DMEM plus 100 nM J147 or fresh DMEM plus 50 ng/ml NGF. Both conditioned medium from J147 treated cells and NGF promoted neurite outgrowth, while the other conditions did not. This effect was reduced by anti-NGF anti-sera. **(H) **BDNF is a target gene of CREB and levels of phosphorylated CREB are significantly increased in the entorhinal cortex by three months of treatment with J147 in these aged huAPP/PS1 mice. Two tailed *P-*values, *P <0.05, **P <0.01, and ***P <0.001. All data shown are means ± SD, N = 10 to 11 per group.

In the AD reversal experiment, J147 treatment increases both proNGF and mature NGF in the hippocampus compared to control huAPP/PS1 mice with the ratio of proNGF to the mature form being decreased significantly upon J147 treatment (Figure 3A, B). In human and rodent brains, proNGF (40 kDa) is the predominant form. A reduction in the ratio of pro- to mature NGF with J147 treatment is important given that proNGF is elevated in AD [66-68], and may contribute to neuronal degeneration [67]. The precursor of BDNF, proBDNF, is secreted and is processed extracellularly to produce mature BDNF [69-71]. Following treatment of huAPP/PS1 mice with J147 late in the course of the disease, there is an increase in both proBDNF and mature BDNF protein levels in the hippocampus, but the ratio of pro- to mature BDNF is decreased with J147 treatment (Figure 3C, D).

Homer 1, an actin binding protein that is induced by BDNF [72], belongs to a family of scaffolding proteins that localize at the postsynaptic density (PSD) [73,74] and is believed to play a critical role in signal transduction, synaptogenesis and receptor trafficking at synapses [75]. Given the increase in BDNF in the hippocampus of J147-treated mice, we investigated the effect of J147 on Homer-1 levels. Figure 3E demonstrates that treatment with J147 significantly increased levels of this protein in aged AD mice.

*Egr3*, which belongs to a family of immediate early genes known as early growth response (Egr) genes, is another target gene for BDNF. Egr proteins play a role in the regulation of synaptic plasticity, learning and memory [76,77] and Egr3 in particular is very important for the processing of both short term and long term hippocampal dependent memory [78]. Figure 3F shows that J147 significantly increased the level of Egr3 in the hippocampus of J147-treated aged AD mice compared to control AD mice.

Additional support for an effect of J147 on neurotrophic pathways came from a study with HT22 cells. HT22 is a nerve cell line derived from mouse brain and is widely used to study nerve cell physiology [22,23]. To examine the effect of J147 on gene expression, a DNA microarray study probed expression of over 34,000 named mouse genes. J147 increased transcription factor *Egr3 *mRNA expression 8-fold following one hour of treatment, while *Ngf *mRNA was up-regulated 2.8-fold (Table 1). HT22 cells do not make BDNF. Also up-regulated was mRNA from another member of the Egr family, *Egr1*, with a 2.5-fold increase and mRNA from a member of the Spred family, *Spred2*, with a 2.7-fold increase. J147 did have an effect on other genes and the Gene Chip data are accessible through GEO Series accession number GSE45534 [26].

**Table 1:** Top up-regulated genes one-hour treatment J147

Gene symbol	Gene title	Entrez gene	Fold change: J147 (1 hr) vs Control
***Egr3***	early growth response 3	13655	8.0
***Ngf***	nerve growth factor	18049	2.8
***Spred2***	sprouty related, EVH1 domain containing 2	114716	2.7
	No title (chr8:13161324 to 13161817)		2.6
***Egr1***	early growth response 1	13653	2.5

DNA microarray was used to evaluate changes in gene expression following one hour of treatment of HT22 neuronal cells with 10 μM J147. The top five genes showing increases in mRNA expression are shown in this table with *Egr3 *and *NGF *being the top two of over 34,000 named mouse genes probed.

To determine the biological consequences of J147 induction of neurotrophin expression, it was asked if conditioned media (CM) prepared from HT22 cells treated with J147 could stimulate neurite outgrowth in PC12 cells. Both conditioned medium from J147 treated cells (Figure 3G, middle right panel) and NGF treated cells promoted neurite outgrowth (Figure 3G, top right panel), while control medium did not (Figure 3G, top and middle left panels). This effect was reduced by anti-NGF anti-sera suggesting that J147 releases neurotrophins with an effect on neurites similar to those released by NGF (Figure 3G, bottom panels). N2 media supplement, which contains transferrin, was added to this experiment to prevent a non-specific protein-mediated effect of antibody on neurite outgrowth.

*BDNF *is a target gene of the cyclic AMP response element binding protein (CREB). Following neuronal stimulation the phosphorylation and subsequent activation of CREB is increased. Furthermore, it is this activity-dependent increase that is believed to facilitate the transcription of proteins required for learning and memory [79,80]. CREB phosphorylation is also thought to limit inflammation [81]. Treatment of aged, transgenic huAPP/PS1 mice with J147 significantly increased the amount of phosphorylated CREB in the entorhinal cortex of these mice (Figure 3H) but not in the hippocampus (data not shown). The entorhinal cortex is one of the first areas to be affected in AD and is the main connection between the hippocampus and neocortex playing an important role in spatial memory [82]. The entorhinal cortex contains the highest level of cholinergic innervations [83] and in AD up to 80% of cholinergic axons can be depleted [84]. Conner and colleagues [85] showed that NGF modulates cholinergic neuronal morphology and postulated that NGF acts by strengthening the cholinergic projections to the hippocampus and cortical areas, which consequently may alter neuronal plasticity and lead to improved memory. Therefore, the effect of J147 on the cholinergic system was investigated using the muscarinic receptor antagonist, scopolamine, which decreases central cholinergic neuronal activity.

### J147 and donepezil in a scopolamine induced model of memory impairment

Cholinergic neurons are among the first to be lost in AD [86,87] and acetylcholine is the therapeutic target for most FDA approved drugs for AD [88,89]. Since J147 increases NGF and NGF is a trophic factor required for cholinergic neurons, we asked if J147 is effective in an assay dependent upon cholinergic transmission. Scopolamine-induced memory impairment in rodents is a well established model of memory dysfunction based upon acetylcholine metabolism [90]. The available data suggest that reversal of scopolamine-induced cognitive impairment is a viable model for predicting pharmacodynamic signals of cognition enhancing compounds in animals [91]. The acetylcholinesterase inhibitor, donepezil, which transiently improves cognition in AD, reverses the cognitive impairment induced by scopolamine in both humans and animals [92,93]. We compared J147 to donepezil in the scopolamine-induced memory impairment model using the same cognitive behavioral assays that were used for reversal of memory impairment in old AD mice. Mice were given J147 alone, donepezil alone and J147+donepezil in food before the cognitive behavioral assays, including the Y-maze, a two-day water maze and fear conditioning were done in the presence of scopolamine treatment. These assays have previously been used to probe deficits with scopolamine treatment [94-97].

**Figure 4:**
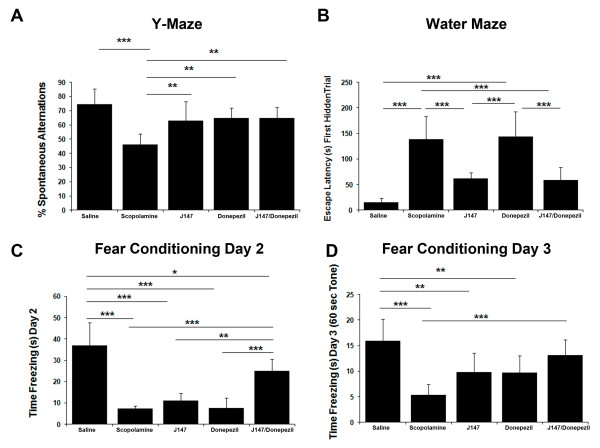
**J147 and donepezil in a scopolamine induced model of memory impairment**. C57BL6/J male mice aged eight weeks were assigned to five groups, 12 mice per group, and treated with compounds in their food for two weeks. Groups included J147 (200 ppm), donepezil (20 ppm), combination of J147 and donepezil (200 ppm and 20 ppm, respectively) and two control groups. Memory impairment was induced with scopolamine at 1 mg/kg 30 minutes before behavior testing in all groups except one of the control groups, which received saline as a control. Mice were analyzed for working memory by the Y-maze, for spatial memory by the two-day water maze, and hippocampal dependent memory by fear conditioning. **(A) **Scopolamine significantly decreased the percent of spontaneous alternations made by mice compared to saline-injected control. Treatment with J147 and donepezil alone, as well as the drugs together, prevented this decrease. **(B) **Treatment with scopolamine significantly increased the time taken to find the platform compared to saline controls in the two day water maze. J147 prevented the loss in spatial working memory, whereas donepezil did not. **(C) **On Day 2 of the fear conditioning assay the amount of time freezing in response to the environment is measured as contextual memory. Scopolamine significantly decreased the freezing response and while J147 did increase the freezing response compared to scopolamine it was not significant. However the J147 and donepezil together did appear to have a synergistic effect. **(D) **On Day 3 the amount of time freezing in response to the tone is measured as emotional memory. Scopolamine significantly decreased the freezing response. While J147 and donepezil alone did increase the freezing response compared to scopolamine, it was not significant. However, J147 and donepezil together had a synergistic effect with the combination significantly increasing the freezing response compared to scopolamine control. One-way ANOVA with Tukey *post hoc *test, *P*-value, *P <0.05, **P <0.01, and ***P <0.001. All data shown are means ± SD, N = 7 to 8 per group.

In all of the assays, mice received an i.p. injection of scopolamine (1 mg/kg) 30 minutes before testing, while one of the groups on the control diet received an i.p. injection of saline. Mice were tested in the Y maze cognitive behavioral assay first. The spontaneous tendency to alternate free choices in entering the three arms of the Y maze is a measure of short-term working memory [20,21]. Scopolamine-treated mice made a lower percentage of spontaneous alternations (defined as consecutive entries into the three different arms) in this test compared to saline controls and all three treatment groups returned this phenotype almost to control levels (Figure 4A). These data indicate that J147, donepezil and J147+donepezil all improve short-term working memory.

Next, mice were observed in the two-day water maze behavioral assay which involves training mice on Day 1 with a visible platform and visual cues and hiding the platform on Day 2. The scopolamine-treated mice took a considerable amount of time to find the hidden platform compared to saline controls (Figure 4B). Both J147 and the combination of J147+donepezil rescued this deficit in spatial memory created by scopolamine to a similar extent, but donepezil alone failed to rescue the deficit (Figure 4B).

Lastly, mice were tested in the fear conditioning behavioral assay. Mice were trained on Day 1 to associate their environment with an aversive stimulus (a foot shock). The amount of time spent freezing in response to the environment is measured on Day 2 as hippocampal dependent memory, whereas the amount of time spent freezing in response to the tone on Day 3 is related to hippocampus and amygdala function. The scopolamine-treated mice spent significantly less time freezing than saline controls in response to the context indicating they did not remember the environmental context (Figure 4C). While J147 treatment did appear to slightly improve contextual memory compared to scopolamine, it was not significant (Figure 4C). Donepezil did not improve memory with the mice showing freezing times similar to scopolamine (Figure 4C). However, the combination of J147+donepezil significantly improved memory compared to scopolamine suggesting a synergistic effect of the two compounds in this assay of hippocampal-dependent associative memory (Figure 4C). Scopolamine-treated mice also spent significantly less time freezing in response to the tone compared to saline controls (Figure 4D). J147 treatment as well as donepezil treatment appeared to rescue the phenotype although neither effect was significant (Figure 4D). However, the combination of J147 and donepezil significantly reversed the memory deficit caused by scopolamine treatment (Figure 4D).

### Pharmacology, safety and target screening of J147

**Figure 5:**
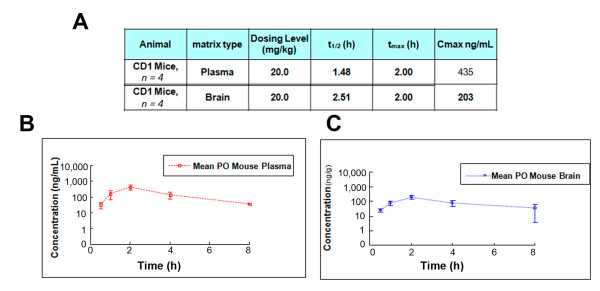
**Pharmacokinetic profile of J147 in mice**. **(A) **The pharmacokinetic profile of J147 in mice was done by Pharmaron (Louisville, KY, USA). Plasma **(B) **and brain **(C) **distribution of J147 was plotted as a function of time following or per oral (PO) administration at 20 mg/kg.

The pharmacokinetics of J147 in the mouse brain and plasma were evaluated using standard procedures. Blood and brain distribution of J147 following per oral (PO) administration was plotted for mice as a function of time (Figure 5). The half life of J147 was calculated at 1.5 hrs in plasma and 2.5 hrs in brain (Figure 5B and 5C, respectively). The bioavailability of J147 following oral administration was calculated at 28%. The safety of J147 was evaluated using acute toxicity, the hERG assay, CYP450 3A4 assays and the Ames test, all of which were negative. Rats and mice received 2 g/kg of J147 to probe acute toxicity, yielding negative results (done by Absorption Systems (San Diego, CA, USA). In contrast, the oral LD_50 _in mice of the widely prescribed donepezil is 45 mg/kg (Pfizer, MSDS, Pfizer Inc, NY, NY, USA). A CeeTox assay also demonstrated "Safe" up to 90 mM J147 plasma concentration (done by CEETOX, Inc., Kalamazoo, MI, USA), and when J147 was fed at 10 mg/kg/day throughout pregnancy to mice, offspring were normal. MDRI-MDCK brain transport rates classified J147 as "High" (done by Absorption Systems), and there was moderate and symmetrical penetration in the Caco-2 assay (Absorption Systems). Additional de-risking and target identification screens were carried out at 10 μM J147 by various CROs (Table 2). No significant reproducible inhibition above 60% was observed except for monoamine oxidase B (MAO B) and the dopamine transporter. Dose response curves were done for each, yielding EC_50_s of 1.88 μM and 0.649 μM, respectively. The EC_50 _of J147 is less than 100 nM in neuroprotection assays [7]. Inhibition of the dopamine transporter can lead to addiction, but no addiction to J147 was observed in mice (study conducted by Dr. Amanda Roberts, Scripps Research Institute). All original data are available upon request.

**Table 2:** Screening for target and off target effects

Screen	Company
Lead Profile Screen (60 CNS receptors/transporters)	Ricerca-MDS
Protein Kinase Screen (50 kinases - activity)	Nova Screen
Protein Kinase (352 kinases - binding)	Ambit
All phosphatases (Tyr and Ser/Thr)	Caliper
Known proteases	Caliper and Ricerca
Deubiquitinases, Esterases, Secretases, Caspases	Caliper
5-, 12-, 15-LOX, COX-1 and 2, NADPH oxidase	MDS
Deacetylase, Sirtuin SIRT-1, -2, -3, Proteasome, MAO A and B	Ricerca
All phosphodiesterases and 5-CYPs	MDS

Various CROs screened a wide variety of potential targets at 10 μM J147 with no significant reproducible inhibition above 60% except for monoamine oxidase B (MAO B) and the dopamine transporter.

## Discussion

The broad neuroprotective activity of the potent, orally active compound J147 was initially described in a double transgenic AD mouse model (APP/swePS1ΔE9). In this model, J147 maintained learning and memory, as well as synaptic proteins, and reduced biochemical markers of inflammation and soluble Aβ levels [7]. Over 200 compounds appear to alter Aβ metabolism or behavioral deficits in AD transgenic mice [30] but none have translated into AD therapeutics [98]. The reason for the lack of translation may be that many of these compounds are only effective when administered before pathology is present; yet, in humans, pathology is usually quite advanced at diagnosis and treatment.

To test the efficacy of J147 in a much more rigorous preclinical AD model, we treated mice using a therapeutic strategy more accurately reflecting the human symptomatic stage. Another mouse preclinical screening trial targeted the symptomatic stage (between 9 and 11 months) [44] and a study with a plant extract [99] used 23- to 24-month-old mice, but to our knowledge no chemically defined AD drug candidate has been tried at the more pathologically advanced stage used in this study. Our strategy involved a 3-month J147 treatment in huAPP/PS1 transgenic AD mice aged to 20 months, an age in which severe behavioral deficits and AD pathology manifest [10]. The goal was to investigate the ability of J147 to rescue cognitive impairment at a late stage in the disease as an indicator of its potential to rescue cognitive impairment in humans with AD. We report here a reversal of cognitive impairment in aged huAPP/PS1 mice by J147 and provide evidence that these beneficial effects are due to the ability of J147 to normalize several different aspects of AD pathology, likely through the up-regulation of the neurotrophin pathway.

Tests that assess distinct aspects of memory can be performed in rodents. Spatial memory is assessed using the Morris Water Maze [100] and hippocampal-dependent associated memory is analyzed by using a fear conditioning assay [101]. In addition, the anxiety response of rodents can be measured using the elevated plus maze, an assay in which AD mice show a disinhibition phenotype [37,38]. In the AD reversal treatment strategy described here, J147 significantly improved several different aspects of memory affected in AD, including spatial memory, a disinhibition phenotype and hippocampal dependent associative learning (Figure 1).

J147 treatment also resulted in a significant effect on Aβ metabolism, reducing levels of soluble Aβ without an effect on plaque density or size (Figure 2). This finding is important considering the accumulating evidence that soluble Aβ directly causes cognitive dysfunction [102,103] and the fact that improved cognition in 3xTg AD mice can manifest without a reduction in Aβ plaque load [104]. Our data (Figures 1 and Figure 2) support this hypothesis. In addition, Zhang and colleagues observed that soluble Aβ is responsible for learning and memory deficits in the huAPP/PS1 mice used in these studies [46]. Our data suggest that the reduction in soluble Aβ levels in the hippocampus of treated, aged AD mice compared to control AD mice by J147 is due to an effect on the APP processing pathway as J147 decreased the protein level of the BACE enzyme leading to an increase in APP levels (Figure 2D, E).

J147 treatment of aged huAPP/PS1 mice increases the expression levels of several proteins involved in neurotrophin signaling. Members of the neurotrophin family maintain neuronal survival, axonal guidance and cell morphology and are key players in cognition and memory formation [55,56]. Neurotrophic factors are perturbed in AD and unevenly distributed due to impairment in axonal transport [105]. It is this imbalance in the AD brain that leads to the observed increase in proNGF in the hippocampus where it is synthesized [106,107] and its reduction in the basal forebrain [108,109]. NGF maintains and regulates the cholinergic phenotype of basal forebrain neurons [110,111]. Figure 3B shows that J147 decreases the ratio of pro- to mature NGF in the hippocampus, which is important given that proNGF is elevated in AD brain [66-68] and may lead to neuronal degeneration [67]. J147 may act to restore the balance between pro- and mature NGF which could allow transport of NGF to the basal forebrain neurons. Results from *in vitro *experiments with the neuronal HT22 cells also suggest an effect of J147 on NGF synthesis and secretion. J147 treatment of HT22 cells for one hour increased NGF mRNA by 2.8-fold in a DNA microarray experiment (Table 1) and conditioned medium from J147 treated HT22 cells stimulated neurite growth in PC12 cells in a NGF-dependent manner (Figure 3G).

*BDNF*, a target gene of CREB, is reduced with age and in the AD brain [112] and is required for normal cognitive function [113]. We previously demonstrated that J147 up-regulates the BDNF pathway in huAPP/PS1 mice following seven months of treatment, and here we show that even when administered at a stage when pathology is advanced, J147 can significantly increase two factors critical for memory formation: CREB phosphorylation and BDNF expression (Figure 3H and 3C, respectively). An increase in both pro- and mature BDNF suggests an increase in BDNF synthesis and secretion from neurons while the decrease in the ratio of pro- to mature BDNF (Figure 3D) suggests higher levels of secreted BDNF. Further evidence for the up-regulation of BDNF signaling comes from the finding of increased levels of Homer-1 and Egr-3 (Figure 3E, F), which are genes activated by BDNF. Thus the phosphorylation of CREB by J147 could increase the levels of BDNF, which consequently may increase BDNF responsive proteins. Hippocampal levels of both BDNF and NGF have been correlated with cognitive performance in animal models [114,115] and administration of these neurotrophic factors reduces memory loss in aging or animal models of AD [116,117], further substantiating their importance for memory. The effect of J147 on both NGF and BDNF levels reported here, as well as its effect on BDNF target genes, may explain the memory deficit reduction observed in the aged huAPP/PS1mice treated with J147 (Figure 1).

Neurotrophic factors have been pursued as appealing candidates for the treatment of neurodegenerative diseases, neuropathies and peripheral nerve injury [118,119]. However, delivering growth factors to the brain has proved difficult and risky for patients with significant side effects observed [120], such as sprouting of sensory and sympathetic neurons [121], and neuropathic pain [120]. Treatment strategies involving neurotrophic factors are now based on the transfer of genes, molecules or cells into the nervous system [122]. NGF is in fact viewed as a viable target for AD clinical trials with one group investigating NGF *ex vivo *gene delivery in a Phase 1 trial with human patients aimed at stimulating cholinergic function and improving memory [123]. This small study found improvement in the rate of cognitive decline but the procedure requires delivery directly into the brain. Perhaps, a molecule that could stimulate neurotrophic factors, such as NGF *in vivo*, would be more efficient, more safe and cost effective than gene delivery directly into the brain.

Scopolamine is a well known competitive muscarinic receptor antagonist that causes reproducible, transient impairments across multiple cognitive domains in healthy animals and non-diseased humans by decreasing central cholinergic neuronal activity [124,125]. The ability of compounds to reverse scopolamine-induced cognitive impairment is used as a model for demonstrating drug target engagement and cognitive enhancement in both humans and animals [91]. In this study, we utilized the scopolamine model to compare J147 to donepezil, the currently most widely used AD drug. Three different behavioral assays, Y-maze, two-day water maze and fear conditioning, which have previously been used to assay deficits with scopolamine treatment [94-97] compared the ability of compounds to reverse cognitive impairment induced by scopolamine. All three assays demonstrated cognitive deficits with scopolamine treatment compared to saline controls (Figure 4A-D). Results from the treatment groups revealed that all three groups, J147, donepezil and J147+donepezil, rescued the decrease in spontaneous alternations induced by scopolamine demonstrating an improvement in short-term working memory (Figure 4A). J147 and J147 + donepezil rescued the deficit in spatial navigational memory caused by scopolamine in the water maze but donepezil alone failed to rescue the deficit (Figure 4B). In the fear conditioning paradigm, only the combination of J147 and donepezil rescued the hippocampal-dependent deficit in contextual and cued fear conditioning, suggesting the compounds' synergistic effect for this particular type of memory (Figure 4C, D). Demonstration of synergism between the two compounds may be important for clinical trials as the majority of patients recruited to a trial will likely already be on donepezil treatment.

The pharmacokinetic properties of J147 after a single dose of 20 mg/kg in mice show brain levels of about 600 nM at 2 h, over 10-fold above its EC_50 _in some cell culture neuroprotection assays [7], with a brain to blood ratio of approximately 0.5. The bioavailability of J147 following PO administration in mice was calculated at 28%. Positive data for blood brain barrier (BBB) penetration (High) were also obtained for J147 using the MDCK-MDRI cell culture model for assaying the BBB penetration potential of drugs. Acute toxicity studies showed no oral toxicity of J147 in mice at the maximum testable dose of 2 gm/kg and other safety tests including hERG, CYP450 inhibition and Ames were also negative which further supports the safety of J147. In the search for the target of J147, many potential off target effects have been ruled out (Table 2), once again suggesting the safety of J147. The data collected to date suggest that J147 has good pharmacokinetic and safety profiles.

## Conclusions

In conclusion, the range of biological activities of J147 relevant to human AD is quite extensive. When administered in food at a stage when pathology is advanced in 20-month-old transgenic AD mice, J147 rescued the severe loss of cognitive function, reduced soluble levels of Aβ and increased neurotrophic factors essential for memory. When compared to donepezil in memory assays, J147 performed equally well or superior, and even showed synergistic effects in the fear conditioning assay. In addition, J147 has good medicinal chemical and pharmacological properties for a CNS drug, has few off-target effects and is orally active [7]. Thus, J147 is an exciting, new compound with strong potential to be an AD therapeutic by slowing disease progression through neuroprotection as well as providing rapid cognition benefits by reversing cognition deficits following short-term treatment. These dual attributes improve the chances for success in clinical trials as a disease-modifying drug, and seems ideally suited for rapid progress through the new FDA guidelines for AD trials [126]. We hypothesize that the mechanism of action of J147 is related to its ability to increase the levels of BDNF and NGF, and studies are being conducted to identify its molecular target.

## Abbreviations

Aβ: beta-amyloid; AD: Alzheimer's disease; APP: amyloid precursor protein; BACE: β-secretase; BBB: blood brain barrier; BDNF: brain derived neurotrophic factor; CM: conditioned medium; CNS: central nervous system; CREB: cyclic AMP response element binding protein; CROs: contract research organizations; CS: conditioned stimulus; DMEM: Dulbecco's Modified Eagle's medium; Egr: early growth response; ESI: electrospray ionization; Hz: Herz; i.p.: intraperitoneal; KPBS: sucrose-potassium-PBS; LCMS: liquid chromatography-mass spectrometry; LTP: long term potentiation; MAO B: monoamine oxidase B; MS: mass spectrometry; NGF: nerve growth factor; NMR: nuclear magnetic resonance; PBS: phosphate-buffered saline; ppm: parts per million; PSD: postsynaptic density; TBAB: tetra-*n*-butylammonium bromide; TLC: thin layer chromatography; TMS: tetramethylsilane; US: unconditioned stimulus.

## Competing interests

The Salk Institute is applying for patents on J147. The authors declare no competing financial interests.

## Authors' contributions

All authors contributed to the manuscript. MP and DS designed the experiments and wrote the manuscript. MP and RD carried out animal cognitive behavioral assays. MP performed all Western blots, ELISAs and immunohistochemistry. DS carried out cell culture experiments and JLE performed DNA microarray assay and corresponding data analysis. CC made the J147 and donepezil compounds used in the study. All authors read and approved the final manuscript.
